# Effects of Aloe Vera and Guava Leaf Extract on Threadfin Bream Surimi Gel: Physicochemical, Textural and Rheological Properties

**DOI:** 10.3390/gels12080728

**Published:** 2026-08-16

**Authors:** Staphny Snowy Dsouza, Umesh Patil, Avtar Singh, Natchaphol Buamard, Mallikarjun Chanchi Prashanthkumar, Hui Hong, Bin Zhang, Yadong Zhao, Soottawat Benjakul

**Affiliations:** 1 International Center of Excellence in Seafood Science and Innovation (ICE-SSI), Faculty of Agro-Industry, Prince of Songkla University, Hat Yai 90110, Songkhla, Thailand; 6811030022@psu.ac.th (S.S.D.); umesh.p@psu.ac.th (U.P.); avtar.s@psu.ac.th (A.S.); natchaphol.b@psu.ac.th (N.B.); mallikarjun.chanchi@gmail.com (M.C.P.); 2 Faculty of Agricultural Technology, Songkhla Rajabhat University, Khao-Rupchang, Muang 90000, Songkhla, Thailand; 3 Beijing Laboratory for Food Quality and Safety, College of Food Science and Nutritional Engineering, China Agricultural University, Beijing 100083, China; hhong@cau.edu.cn; 4 Key Laboratory of Health Risk Factors for Seafood of Zhejiang Province, College of Food Science and Pharmacy, Zhejiang Ocean University, Zhoushan 316022, China; zhangbin@zjou.edu.cn (B.Z.); zhaoyd@zjou.edu.cn (Y.Z.); 5 Department of Food and Nutrition, Kyung Hee University, Seoul 02447, Republic of Korea

**Keywords:** surimi gel, threadfin bream, aloe vera powder, guava leaf extract, polyphenols, acemannan, rheology

## Abstract

Polyphenol-mediated protein interactions offer a promising approach for improving surimi gel networks. Nevertheless, the comparative and combined effects of polyphenol-rich plant extract and polysaccharide-based additives on gel properties remain insufficiently explored. Therefore, individual and combined effects of dechlorophyllized guava leaf extract (DGLE), a source of polyphenols and aloe vera powder (AVP), an acemannan-rich polysaccharide, on the physicochemical, textural and rheological properties of threadfin bream (*Nemipterus* spp.) surimi gel were investigated. DGLE obtained by ultrasonication followed by dechlorophyllization exhibited high total phenolic content (439.35 ± 4.72 mg GAE/g dry extract) and total flavonoid content (666.66 ± 60.52 mg QE/g dry extract). LCMS/MS-QTOF profiling tentatively identified 12 phenolic compounds, including ellagic acid, catechins, quercetin glycosides, EGCG, etc. Surimi gel incorporated with 0.2% AVP and 0.05% DGLE individually achieved the highest breaking force and deformation with minimum expressible moisture content, though whiteness decreased with increasing concentrations. SDS-PAGE and FTIR analyses confirmed polyphenol-protein interaction and acemannan-gel network association. Gel with 0.2% AVP alone showed the highest hardness, gumminess, chewiness and elevated *G*′, while 0.05% DGLE alone yielded the highest springiness and cohesiveness, consistent with dense cross-linking observed in SEM (*p* < 0.05). AVP combined with DGLE exhibited a reduced gel-strengthening effect, especially at increasing DGLE concentrations. Thus, DGLE alone at 0.05% improved surimi gel properties. This finding could provide insight into the use of DGLE as a natural additive to improve surimi gel.

## 1. Introduction

Global seafood consumption is projected to increase by 19 million tonnes by 2032, driven by aquaculture expansion, rapid urbanization, rising incomes, and shifts in dietary preferences [1]. With the expanding market, demand for convenient, nutritious and safe processed seafood products has accelerated growth of the ready-to-eat (RTE) food sector [2]. Surimi-based products occupy a significant share of this market owing to their affordability, nutritional value and versatile textural properties [3].

Surimi production was traditionally developed in Japan through repeated washing of fish mince and de-watering to remove water-soluble components, thereby concentrating the myofibrillar proteins (MPs) responsible for heat-induced gelation [4]. Temperate species such as Alaska pollock, arrowtooth flounder, Pacific whiting and blue whiting have traditionally been employed for surimi production. However, tropical species such as lizardfish, bigeye snapper and particularly threadfin bream have become increasingly important, especially in Southeast Asia and Thailand owing to their lean muscle, high MP content, strong gel-forming ability, white color and elastic texture [5,6]. Despite these favorable qualities, threadfin bream surimi is susceptible to the modori phenomenon, a heat-induced gel degradation occurring between 50–70 °C [7]. At these temperatures, endogenous serine and cysteine proteinases are activated and promote autolysis of MP, particularly degradation of the myosin heavy chain (MHC), resulting in a weakened gel network with reduced strength and elasticity [8]. Therefore, the incorporation of additives, such as polyphenols, protein cross-linkers (e.g., transglutaminase), or protease inhibitors (e.g., egg white, soy protein isolate), to inactivate indigenous proteases has been widely used to improve surimi gel quality [9].

Polyphenols and polysaccharides from plant-derived sources have gained increasing interest for improving the gelling properties of surimi. Polyphenols enhance gelation in surimi primarily through non-covalent interactions involving hydrogen bonding and hydrophobic interactions and covalent cross-linking mediated by the oxidation of phenolic compounds to quinone intermediates, which then react with sulfhydryl (-SH) and amino acid residues (cysteine, tryptophan, lysine) to form irreversible C-N and C-S bonds [10]. These interactions collectively strengthen the three-dimensional gel matrix and can simultaneously inhibit protease activity by modifying the active sites of endogenous enzymes [9]. Surimi gel-forming efficacy has been achieved by adding various polyphenolic compounds, including hyperoside [11], and polyphenol-rich plant by-products such as pomegranate peel [12], apple, tea, and olive leaf powder [10].

Guava leaves are known for containing a higher concentration of phenolic compounds than the fruit, seeds or stem bark. The leaves are rich in quercetin, rutin, ellagic acid, catechin, kaempferol, and hyperoside. Those compounds possess antioxidant, anti-inflammatory, antibacterial, antihyperglycemic and anticancer properties [13]. Ultrasound-assisted extraction has been identified as the most efficient method to recover bioactive compounds from guava leaves, and ethanolic concentrations between 60% and 80% have been documented to optimize the extraction of phenolic compounds [14,15]. Dechlorophyllized guava leaf extract (DGLE) has not been previously evaluated for surimi gelation and represents a clean-label, sustainable, plant-derived additive aligned with current trends toward natural food ingredients.

Apart from plant polyphenols, polysaccharides have been widely employed to enhance surimi gelation through several mechanisms, including protein network reinforcement and water retention improvement [16]. Polysaccharides act as active fillers within the MP matrix, contribute to the structural compaction of the gel structure and immobilize water through extensive hydrogen bonding [8]. In addition, polysaccharides can improve gel elasticity and stability by strengthening protein–polysaccharide interactions and reducing moisture migration during thermal processing [17]. Polysaccharides such as κ-carrageenan [3], chitooligosaccharide [18], fucoidan [16], tamarind kernel powder [6], gellan [19] and konjac glucomannan [20] have been utilized in the surimi system.

Aloe vera (*Aloe barbadensis* Miller) powder (AVP) is another promising plant-derived food ingredient. The functional properties of AVP are attributed to acemannan, an acetylated glucomannan responsible for the characteristic mucilaginous nature of raw aloe vera [21]. Acemannan is a polysaccharide composed of monomers such as β-(1 → 4)-D-mannose and 1 β-(1 → 4)-D glucose at a ratio of 3:1, with side chains of α-(1 → 6)-D galactose and *O*-acetyl groups [21]. The abundance of hydroxyl and acetyl groups in acemannan enables it to interact with water molecules and proteins through hydrogen bonding, thereby contributing to improved water-holding capacity and gel-network stability. Kim et al. [22] reported enhanced gel properties in surimi via addition of AVP combined with red ginseng.

Although the individual application of polyphenols or polysaccharides in surimi gelation has been reported, the use of DGLE and AVP, either individually or in combination, in surimi gel systems remains unexplored. The combined incorporation of AVP and DGLE into surimi gel might create a ternary polyphenol-protein-polysaccharide system [23]. The appropriate combination of DGLE and AVP might improve the surimi gel properties. Hence, this study aimed to evaluate the influence of DGLE and AVP individually and in combination on the gel properties of threadfin bream surimi. Novel insight into their individual roles in modulating surimi gel functionality could be gained. The impact of DGLE at varying concentrations in the presence of AVP at the selected content on surimi gel properties was also examined.

## 2. Results and Discussion

### 2.1. TPC and TFC of DGLE

DGLE had a TPC of 439.352 ± 4.72 mg GAE/g freeze-dried DGLE powder and TFC of 666.66 ± 60.52 mg QE/g freeze-dried DGLE powder. TPC obtained in the present study was consistent with the value of 422.84 ± 22.91 mg GAE/g reported by Olatunde et al. [24], who employed similar extraction conditions for DGLE. In contrast, the TPC values reported by Huynh et al. [13] across various extraction methods using guava leaf as raw material ranged between 83 and 127 mg GAE/g of dried leaves, suggesting considerable variation among findings. Those extraction methods include the use of methanol at 65 °C in a rotary shaker at 350 rpm for 6 h, the use of 50% ethanol for 20 min via sonication and the use of 70% ethanol through reflux at 70 °C for 30 min. However, extraction using enzymes such as cellulase, xynalase and β-glucosidase showed a TPC of 438.40 mg GAE/g [13]. The high TPC and TFC values observed in the present study might be due to the use of ultrasonication to aid extraction. Ultrasonication enhances the release of phenolic compounds through acoustic cavitation that disrupts plant cell walls and promotes the diffusion of the constituents into the solvent [14]. Apart from ultrasonication used, the elevated TPC and TFC observed in the present study might be due to the dechlorophyllization. The reduction of chlorophyll from the extract likely led to the increased proportion of phenolic compounds in the extract. Singh et al. [25] demonstrated that aqueous dechlorophyllization by sedimentation significantly increased both TPC and TFC in guava leaf extracts, attributed to the hydrophobic nature of chlorophyll and allowing polyphenols to remain preferentially in the aqueous phase. Lorena et al. [26] reported TFC in the range of 558.06–749.42 µg rutin equivalent/mg extract in the guava leaf decoction, and TFC expressed in terms of catechin equivalent was in the range of 121.43–158.72 µg CE/mg extract. TFC of 401.66 mg QE/g dry extract was observed by Chowdhury et al. [27] in methanolic guava leaf extract. Overall, the differences observed in TPC and TFC across studies were likely influenced by a combination of factors, including plant variety, growing conditions and the extraction technique used [13].

### 2.2. Phenolic Compounds in DGLE

Phenolic compounds tentatively identified in DGLE in both negative and positive ionization modes, analyzed by LC-MS/MS quadruple time-of-flight, are presented in Table 1. Tentative identification was done based on retention time and *m*/*z* values; 12 phenolic compounds were identified. In negative ionization mode, ellagic acid was the most dominant compound with an abundance of 1.690 × 10^7^, followed by (−)-catechin (3.987 × 10^6^) and (−)-epicatechin gallate (3.065 × 10^6^). In positive ionization mode, (+)-catechin was the most abundant (1.427 × 10^7^), followed by (−)-gallocatechin (8.993 × 10^6^) and quercetin (8.189 × 10^6^). The detected compounds, such as (+)-catechin, (−)-catechin, (−)-epicatechin gallate, (−)-gallocatechin, and (−)-epigallocatechin gallate, are the isomers of catechin [28]. Kaempferol, a flavonol, was observed in both ionization modes, with the same retention time of 13.01 min but at *m*/*z* of 285.041 in negative mode and *m*/*z* of 287.056 in positive mode, consistent with the detection of the ions of the same kaempferol molecule [29]. Quercetin was also tentatively identified with an abundance of 8.189 × 10^6^ and a molecular weight of 302.04. It is a commonly present flavonoid in guava leaves [13].

Moreover, quercetin-glycosides were also identified in the extract. Hyperoside (quercetin-3-O-galactoside) was found in negative mode, while guaiaverin (quercetin-3-O-α-L-arabinopyranoside) was detected in positive mode. Glycosidic derivatives of quercetin could help enhance the solubility of the flavanol by providing additional OH-groups and promote H-bonding through their sugar moieties [30]. The tentatively identified phenolic compounds in the present study were in agreement with those reported by Olatunde et al. [24] in DGLE prepared by the sedimentation method. Those phenolic compounds might have contributed to protein cross-linking in the surimi gel network, thus governing gel properties. Yun et al. [31] reported that polyphenols acted as a cross-linker, enhancing the gel strength of minced lamb through covalent bonding.

### 2.3. Effect of AVP, DGLE and Their Combination on Breaking Force (B-F) and Deformation (D-F) of Surimi Gel

B-F and D-F are crucial parameters indicating surimi gel quality and are directly influenced by the type and concentration of incorporated addtive [32]. The effects of AVP or DGLE alone at different levels, or of 0.2% AVP + DGLE at varying concentrations, on B-F and D-F of surimi gel are presented in Table 2. Incorporation of 0.2% AVP resulted in higher B-F and D-F than other samples containing AVP at other levels (*p* < 0.05). Gel containing 0.2% AVP had an increase in B-F and D-F by 33.61% and 24%, compared to those of the control. The addition of AVP above 0.2% resulted in decreases in both B-F and D-F. This enhancement might be attributed to the hydrophilic nature of AVP, which might interact with the hydrophilic domain of myofibrillar proteins. It plausibly occupied interfibrillar voids within the surimi network, thereby reinforcing the gel structure as a filler [3]. Acetylated glucomannan (acemannan), a major polysaccharide present in AVP containing O-acetyl (-OCOCH_3_) groups, might be involved in hydrophobic interaction, while hydroxyl groups participated in dominant hydrogen bonding, collectively strengthening the gel network at the appropriate concentration [3,21]. Yuan et al. [33] reported the reduced hydrophobic interactions and enhanced hydrogen bonding in partially deacetylated konjac glucomannan (DKGM) with sufficient acetyl groups, thereby providing steric hindrance and preventing self-aggregation of DKGM. This phenomenon thus promoted enough stretching to interact with proteins. Partial deacetylation (up to 20%) reported by Minjares-Fuentes et al. [34] might have promoted the balance between hydrophobicity and hydrophilicity, thus enhancing gel strength. Yan et al. [35] found that KGM with a degree of deacetylation of 50.7% was suitable for surimi products. AVP concentrations of 0.05 and 0.1% had minimal effect on B-F and D-F. At higher levels, the decreases in both parameters became more pronounced. With higher levels of AVP, myofibrillar proteins in surimi were diluted. As a consequence, the interaction between muscle proteins became less. This could weaken the strength of the surimi gel. The lowering effect of AVP on D-F was also observed, indicating the lower elasticity of the gel. However, there was no difference in both B-F and D-F between surimi gel samples added with 0.4 and 0.5% AVP (*p* > 0.05). Thus, 0.2% AVP was the optimal level of addition to threadfin bream surimi, as indicated by increased B-F and D-F.

When DGLE at different levels was incorporated into surimi gel, the resulting gel added with DGLE at 0.05% showed the highest B-F and D-F (*p* < 0.05), in which increases of 35.88% and 20.8% were obtained, compared to those of the control. This enhancement is more likely attributed to the protein cross-linking ability of phenolic compounds present in DGLE as identified by LC-QTOF analysis (Table 1). Phenolic compounds plausibly interacted with surimi proteins through both reversible non-covalent bonds, including hydrophobic interactions, hydrogen bonds, and ionic interactions, as well as irreversible covalent bonds. Phenolic hydroxyl groups were plausibly oxidized to quinone intermediates. Those molecules with high electrophilicity might react with thiol (-SH) and amino groups of MHC, collectively strengthening the gel network [36]. Moreover, flavonoids with sugar moieties such as hyperoside (quercetin-3-O-galactoside) and guaiaverin (quercetin-3-O-arabinopyranoside) could provide additional hydroxyl groups from their galactose and arabinose residues, respectively. As a result, the gel strength could be enhanced. The strengthening effect of hyperoside on threadfin bream surimi gel was documented [11]. Balange and Benjakul [36] reported that adding 0.05% oxidized tannic acid to bigeye snapper surimi gel yielded optimal B-F and D-F. In the present study, further decreases in both B-F and D-F were found with increasing concentrations of DGLE up to 0.5%. This might be due to the self-aggregation of polyphenols, thus lowering protein cross-linking capability [37]. Aggregated polyphenols had no binding sites for interacting with surimi protein chains. This was witnessed by the lower B-F and D-F.

When 0.2% AVP was used in combination with DGLE at the selected concentrations (0.05%, 0.1, 0.2%), high B-F and D-F were still obtained in comparison with those of the control (*p* < 0.05). However, B-F of all surimi gels containing both AVP and DGLE was lower than that of the gel added with 0.05% DGLE alone (*p* < 0.05), suggesting a lower effect in the combination of additives. However, Kim et al. [22] demonstrated optimal gel strength of Alaska pollock surimi gel when AVP was incorporated in conjunction with 0.2% red ginseng extract containing mainly ginsenosides. The lowered effect could be due to the different complex interactions in various food systems as reported by Xue et al. [23] for the ternary polyphenol–protein–polysaccharide systems. Polysaccharides and proteins plausibly competed for the same binding sites on polyphenols through non-covalent interactions, potentially forming polysaccharide-polyphenol complexes, since the addition and blending of AVP were done prior to DGLE addition, thereby compromising polyphenol-protein interactions.

In addition, the presence of AVP could interfere with the interaction between proteins chained as bridged by polyphenols in DGLE. The incorporation of DGLE, regardless of concentration, hampered the formation of ordered gel in the presence of AVP. It was noted that the prior mixing of AVP with surimi before addition of DGLE might hinder the subsequent protein cross-linking induced by polyphenols in the DGLE. Therefore, the lower performance of the combination treatment might reflect the crucial impact of mixing sequence rather than an inherent antagonistic interaction.

### 2.4. Effect of AVP, DGLE and Their Combination on Textural Properties

Hardness, springiness, cohesiveness, gumminess, and chewiness are the principal texture profile analysis (TPA) parameters used to evaluate textural properties of surimi gel [38]. The TPA parameters of surimi gel incorporated with AVP, DGLE and 0.2% AVP + DGLE at varying concentrations are presented in Table 3. Among all samples added with AVP at various levels, those added with 0.2% AVP exhibited the highest hardness (11,436 g) (*p* < 0.05). The acemannan in AVP might act as a filler that physically interpenetrates the myofibrillar protein network during thermal gelation, forming a protein-polysaccharide composite network with enhanced compressive resistance [19]. Additionally, the presence of abundant -OH groups could immobilize free water through hydrogen bonding and interact with myofibrillar protein, thus more likely promoting a strong network and hardness [39]. However, surimi gel containing AVP above 0.2% had decreased hardness, with increasing AVP levels. These results implied that the excessive amount of polysaccharide compromised the continuity of the protein gel network, thereby limiting the increase in gel hardness [16]. Springiness of gel samples added with AVP at all levels along with the control ranged narrowly (0.86–0.91). The result was consistent with previous reports on crude fucoidan incorporated surimi, which primarily reinforced compressive resistance rather than elasticity [16]. The samples containing 0.3–0.5% AVP also had decreased cohesiveness (0.75) (*p* < 0.05), but the addition of 0.2% AVP showed an increase in cohesiveness, indicating superior structural integrity under repeated compression cycles. Polysaccharide at high concentration might mediate the disruption of myofibrillar protein aggregation [40]. Gumminess and chewiness showed a similar trend to hardness, as these parameters are mathematically derived from hardness, cohesiveness and springiness [40].

For surimi gel incorporated with DGLE at various levels, the sample added with 0.05% DGLE exhibited the highest hardness and springiness among all DGLE-incorporated samples (*p* < 0.05). The improved hardness was due to the ability of guava leaf polyphenols to interact with myofibrillar proteins through non-covalent hydrogen bonds and hydrophobic interactions, thus promoting protein cross-linking and enhancing gel network formation. The results were consistent with findings of Sharma et al. [12], in which silver carp surimi gel added with pomegranate peel extract had improved hardness and springiness via polyphenol-protein interactions. Notably, the polyphenol-induced improvement in springiness of the gel added with 0.05% DGLE (0.94) was observed, which was higher than that of other samples and the control (*p* < 0.05). This might be due to its low concentration, which did not enhance an excessive protein cross-linking. Therefore, the rigidity was not too high, while providing the elasticity as indicated by increased springiness [11]. Polyphenols in DGLE at low concentrations likely promoted the greater elasticity by cross-linking myofibrillar proteins through reversible non-covalent interactions. Cohesiveness of surimi gel containing DGLE exhibited a concentration-dependent effect, where increasing concentration led to a reduction in cohesiveness. Threadfin bream surimi added with hyperoside formed the weaker gel at higher concentrations, and the resulting gels were easily disrupted upon compression [11]. Moreover, gumminess and chewiness in DGLE-added surimi gel followed a similar pattern observed for hardness.

Although both surimi gels added with 0.2% AVP or 0.05% DGLE individually produced significant textural improvements over the control, the combination of 0.2% AVP and DGLE at different levels displayed varying textural attributes. With increasing DGLE concentration, interactions between AVP polysaccharides and DGLE polyphenols in the surimi gel matrix might have formed. This interaction might hinder polyphenol-protein interaction, and dilution effect on myofibrillar protein by AVP could occur [23]. When such polyphenol-AVP complexes were formed, filler ability of polysaccharide and protein cross-linking were partially suppressed, leading to poorer gel properties. Cohesiveness dropped markedly to 0.64 when 0.2% DGLE was incorporated in the presence of 0.2% AVP, in which structural integrity was disrupted under repeated compression. Gumminess and chewiness followed a similar trend to hardness across all combination treatments. High B-F and D-F observed in surimi gel containing 0.2% AVP and 0.05% DGLE exhibited high hardness, springiness and cohesiveness. Furthermore, Duan et al. [39] found a positive correlation between gel strength and hardness in surimi gel. Therefore, the levels of AVP and DGLE should be optimized to improve gel performance.

### 2.5. Effect of AVP, DGLE and Their Combination on EMC

EMC of a surimi gel indicates the ability of a gel to retain water under external forces [7]. Gels possessing the highest B-F and D-F had the lowest EMC, signifying a fine and ordered gel network, which was able to entrap more water. AVP could hold more water in its network [41]. Surimi gels with the highest B-F and D-F, when added with 0.2% AVP and 0.05% DGLE individually, exhibited lower EMC, and the combinations of AVP and DGLE also had lower EMC than the control surimi gel, as shown in Table 2. However, gel containing 0.2% DGLE and 0.2% AVP showed similar values to that of the control. AVP, with multiple -OH groups, could have contributed to H-bond formation with water molecules, leading to enhanced water retention. The result was consistent with the water-binding role of -OH-rich polysaccharides such as chitooligosaccharide in sardine surimi [18]. Additionally, the presence of acetyl groups in mannans provided steric hindrance between the mannans, thus promoting water solubility as reported by Karimi et al. [42]. Therefore, low EMC was found in surimi gel as acemannan chains were present in AVP. However, the addition of AVP above 0.2% tended to dilute the myofibrillar proteins, thereby reducing the formation of a strong gel network and leading to failure of water retention [10]. Hence, AVP at 0.2% was selected for combination with DGLE at various concentrations.

Gel incorporated with 0.05% DGLE exhibited the lowest EMC (*p* < 0.05) (Table 2). The results were in accordance with Zhou et al. [38], in which protein aggregation induced by epigallocatechin gallate (EGCG) in silver carp surimi gel occurred properly at lower concentrations of 0.02% and 0.04% via covalent non-disulfide bond formation between polyphenols and -SH groups of proteins with the highest water holding capacity. However, the increase in EGCG concentration disrupted the gel network due to poor protein aggregation. Similarly, Singh et al. [11] reported that excessive amounts of hyperoside interfered with the formation of a strong gel network in threadfin bream surimi and obstructed the ability of the gel to retain water.

In general, gels comprising 0.2% AVP and those containing 0.05% DGLE had similar EMC, but their combination with increased DGLE concentration resulted in the increased EMC, indicating a decrease in available binding sites for DGLE polyphenols. Generally, H-bonds might be involved in the dominant interaction with water, as indicated by high water holding capacity [11,18]. An increase in the gel’s EMC with increasing DGLE concentrations might be due to polyphenol self-aggregation at higher concentrations, thereby reducing the available protein cross-linking sites and disrupting the ordered network necessary for water immobilization [12]. Collectively, the structural integrity of the three-dimensional protein network and its water-holding capacity were influenced by both levels of AVP and DGLE incorporated. Overall, the combinations were less effective than the best individual treatment, as combination treatments compromised the water-holding capacity of the resulting threadfin bream surimi gel.

### 2.6. Effect of AVP, DGLE and Their Combination on Whiteness of Surimi

Whiteness is an important quality index for surimi gel and products, which is dependent on the type and quantity of the additive [19]. The whiteness values of gel added with AVP, DGLE and AVP combined with DGLE are presented in Table 4. Incorporation of AVP resulted in a progressive concentration-dependent increase in whiteness. Gel with 0.5% AVP exhibited the highest whiteness value (75.32), while the control (73.69) and all other treated surimi gels showed lower whiteness (*p* < 0.05). The upsurge in whiteness was related to the reduction in yellowness (b*). This was possibly due to the light-scattering ability of AVP, especially in its hydrated form within the surimi gel [6]. Additionally, Duan et al. [43] reported that golden pompano surimi gel with a dense protein network and high water retention exhibited superior light-scattering properties, leading to enhanced whiteness.

In contrast, the incorporation of DGLE significantly reduced the whiteness values, especially with increasing added levels. The decline in whiteness coincided with the rises in a* and b* values. This phenomenon is commonly found in surimi gel incorporated with phenolic-rich plant extracts. The reduction of whiteness in threadfin bream surimi was mainly attributed to the yellowish color of hyperoside or other phenolic compounds [11]. This could be managed by targeting surimi products where color is less critical for their acceptance. Although initial dechlorophyllization of GLE lowered the green discoloration but residual yellow-brown polyphenolic pigments like quercetin were more likely to contribute to the yellowish color of DGLE. Furthermore, during thermal processing, guava leaf polyphenols probably undergo oxidation to form quinone intermediates with brown color [37]. Moreover, the combination of AVP and DGLE could prevent the drastic change in whiteness caused by DGLE polyphenols owing to the light scattering impact of AVP on the gel. Apart from discoloration of surimi gel as induced by DGLE addition, astringent taste could be found in the resulting gel to some extent. However, the level of DGLE was very low, and the interaction with proteins in saliva might be reduced. Therefore, the negligible astringent taste was assumed.

### 2.7. Effect of AVP, DGLE and Their Combination on Protein Pattern

**Figure 1:**
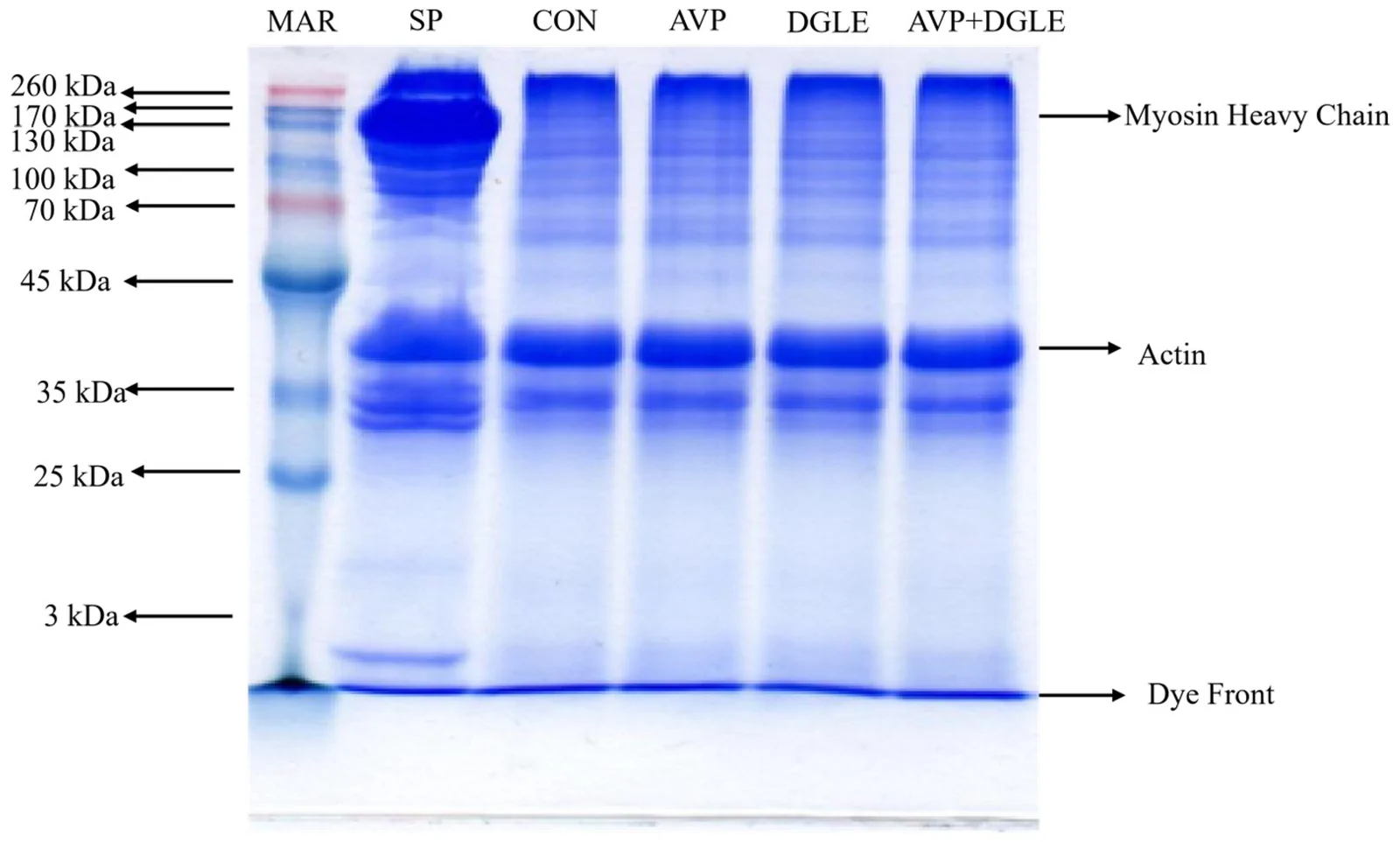
SDS-PAGE protein patterns of threadfin bream surimi paste and gel with aloe vera powder (AVP) (0.2%); dechlorophyllized guava leaf extract (DGLE) (0.05%); with combination (0.2% AVP + 0.05% DGLE); MAR: Long-range protein molecular weight marker; SP—surimi paste; CON—gel without any additives (AVP or DGLE).

SDS-PAGE protein patterns of surimi paste, surimi gel without additives (control), surimi gel with 0.2% AVP, gel containing 0.05% DGLE and their combination (0.2% AVP + 0.05% DGLE) are shown in Figure 1. The protein profiles of surimi paste examined under reducing conditions showed two prominent protein bands, including MHC at ~200 kDa and actin at ~45 kDa. The result was consistent with the protein profile of surimi paste reported in previous studies [3,15]. The tropomyosin band also appeared with the intermediate molecular weight of 30 kDa, reflecting the typical myofibrillar protein composition of fish muscle [44].

In the control surimi gel, MHC band intensity was markedly reduced, signifying the protein cross-linking mediated by endogenous transglutaminase (TGase). This phenomenon was enhanced during setting at 40 °C, facilitating the formation of non-disulphide covalent ε-(γ-glutamyl)lysine isopeptide bonds [19]. The results indicated that TGase activity was prominent in covalent network formation in the control gel. In contrast, the actin band (~45 kDa) was retained with comparable intensity across samples, asserting its unsuitability as a substrate for TGase due to the inaccessibility of reactive glutamine and lysine residues in its molecule [3].

Protein band patterns observed for surimi gel incorporated with 0.2% AVP, or 0.05% DGLE alone and gel containing 0.2% AVP combined with 0.05% DGLE exhibited similar reduced MHC band intensity and retained actin bands to the control. During sample solubilization in the presence of SDS at 85 °C, all non-covalent interactions were destroyed. As a consequence, similar protein patterns were observed across the additive-incorporated gels and the control. The result suggested that the additives at these concentrations mainly contributed to the protein cross-linking mediated by weak bonds or non-covalent bonds [45]. The improved textural properties of surimi gel when added with the additives were mainly attributable to non-covalent network reinforcement, consistent with similar protein patterns in surimi gel with and without additives such as surimi gel incorporated with κ-carrageenan [3] or gellan [42].

### 2.8. Effect of AVP, DGLE and Their Combination on Secondary Protein Structure of Surimi Gel

**Figure 2:**
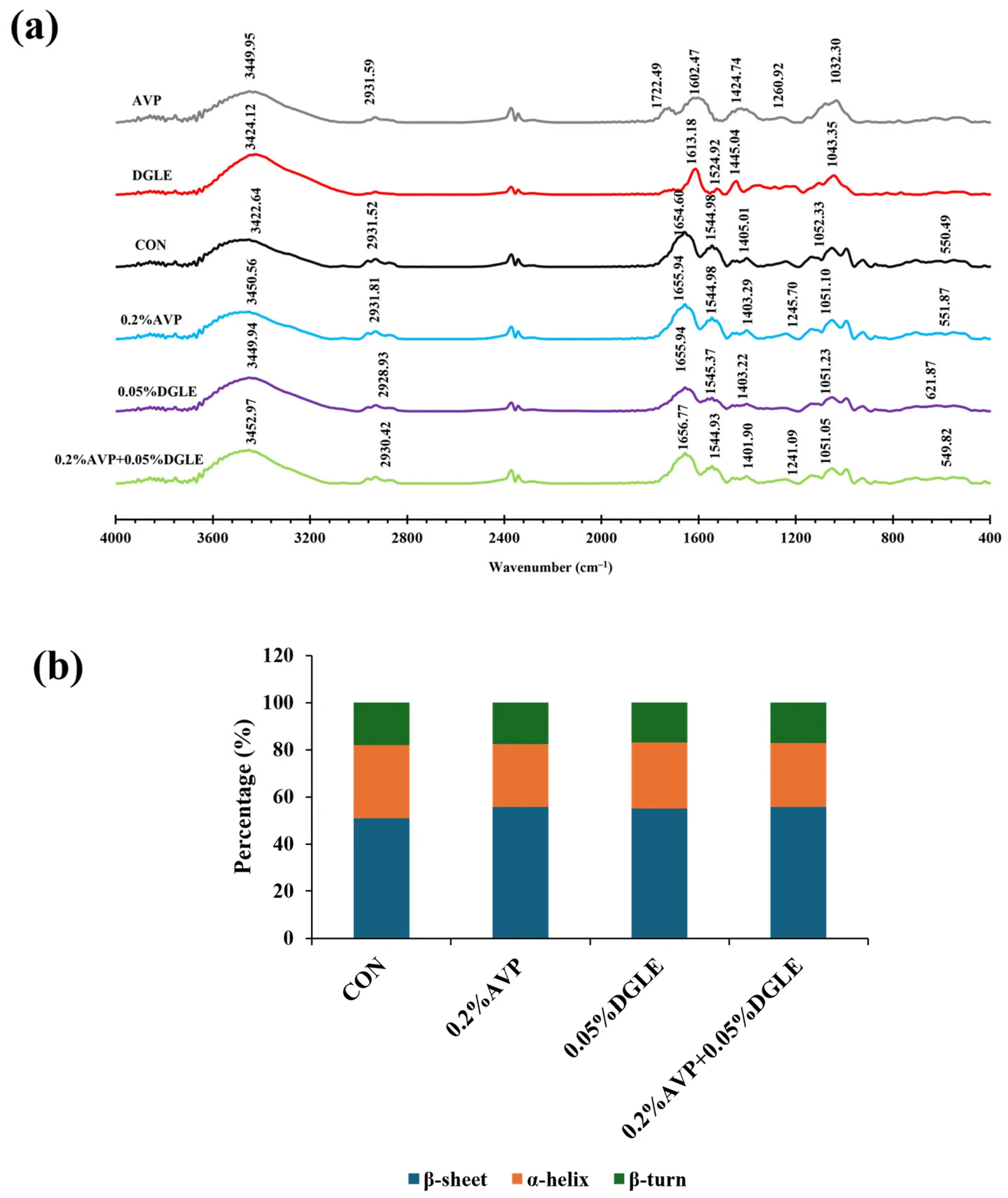
FTIR spectra (**a**) of aloe vera powder (AVP), dechlorophyllized guava leaf extract (DGLE), and threadfin bream surimi gels incorporated with AVP, DGLE and their combination AVP + DGLE in comparison with the control over the range of 400–4000 cm^−1^. Characteristic absorption bands are indicated with their corresponding wavenumber values cm^−1^. Deconvoluted values represent the relative percentage contribution of each sub-band to the total fitted amide I band area (1700–1600 cm^−1^) (**b**); AVP—aloe vera powder; DGLE—dechlorophyllized guava leaf extract; CON—surimi gel without additives; 0.2% AVP—surimi gel incorporated with 0.2% AVP; 0.05% DGLE—surimi gel incorporated with 0.05% DGLE; 0.2% AVP + 0.05% DGLE—surimi gel with 0.2% AVP and 0.05% DGLE.

The FTIR spectra displayed the characteristic functional groups of AVP and DGLE and the effect of those additives on the secondary structure of protein in surimi gel, including gel added with 0.2% AVP, 0.05% DGLE and combined additive (0.2% AVP + 0.05% DGLE as shown in Figure 2a. AVP exhibited a broad -OH stretching band at 3449 cm^−1^ attributable to hydroxyl groups in its carbohydrate monomers, along with -CH stretching at 2931 cm^−1^, a C=O ester band at 1722 cm^−1^ and a C-O-C stretch at 1260 cm^−1^, the typical characteristic of acetyl groups in AVP. Additional absorptions at 1602 cm^−1^ (C-O), 1424 cm^−1^ (symmetric COO-) and C-O-C stretching of glycosidic linkage at 1032 cm^−1^ were consistent with acemannan spectra previously reported [34,46]. The C=O band at 1722 cm^−1^ appeared at a lower wavenumber than the 1760–1740 cm^−1^ range typically reported for intact acetyl esters, while the 1260 cm^−1^ band was also displaced from the 1248 cm^−1^ position. These shifts are consistent with partial deacetylation during the freeze-drying process, which would reduce acetyl ester content and shift the residual band toward lower wavenumbers [34].

DGLE displayed a broad -OH stretching band at 3424 cm^−1^, reflecting the cumulative phenolic and flavonoid hydroxyl groups. The result was consistent with the presence of ellagic acid, quercetin, catechins, EGCG and kaempferol derivatives identified by LCMS/MS QTOF analysis. Aromatic C=C skeletal vibrations of the flavonoid ring system appeared at 1613 cm^−1^ and 1527 cm^−1^, while the band at 1043 cm^−1^ corresponded to C-O-C ether and glycosidic linkages of the flavonoid glycoside fraction such as hyperoside and guaiaverin identified in the present study [27].

The FTIR spectra of all surimi gel treatments and the control exhibited similar absorption patterns, confirming that neither AVP nor DGLE introduced new covalent bonds into the gel matrix. Both additives modulated the positions of pre-existing functional-group bands in the surimi gel system. Amide I (C=O stretching, 1700–1600 cm^−1^) and amide II (N-H bending coupled with C-N stretching, 1600–1500 cm^−1^) bands were identified as primary indicators of proteins. The amide I band is particularly sensitive to conformational changes in myofibrillar proteins with sub-band regions at 1618–1640 cm^−1^, 1640–1650 cm^−1^, 1650–1660 cm^−1^ and 1660–1690 cm^−1^, corresponding to β-sheet, random coil, α-helix and β-turn, respectively [3]. The band near 1050 cm^−1^ is more likely associated with carbohydrate C-O or C-O-C vibrations.

The control gel showed an amide I peak at 1654 cm^−1^. When incorporated with AVP, DGLE or combined AVP and DGLE, slight shifts in the peak were observed at 1655.94 cm^−1^, 1655.94 cm^−1^ and 1656.77 cm^−1^, respectively. Deconvolution of the amide I band as shown in Figure 2b indicated the shift in protein conformation across all surimi gels added with additives in comparison to the control surimi gel. The β-sheet content increased from 50.84% in the control to 55.61%, 55.00% and 55.74% in surimi gels added with AVP, DGLE and combined AVP + DGLE, respectively. The decrease in α-helix and modest decline in β-turn were observed, with random coil content being negligible in all groups. Uncoiling of the α-helix chain during heat-induced gelation and formation of β-sheet structure tended to expose the hydrophobic residues, further enhancing hydrophobic interactions, which could increase the protein aggregation [33].

Incorporation of AVP and DGLE also caused the shift of the -OH/-NH stretching band of the surimi gels. The -NH stretching of control gel shifted from 3422.64 cm^−1^ to 3450.56 cm^−1^, 3449.94 cm^−1^ and 3452.97 cm^−1^ in surimi gels added with AVP, DGLE and combined AVP + DGLE, respectively. This was possibly owing to the interaction between hydroxyl groups of AVP acemannan and DGLE polyphenols [12]. As a result, the interaction between polyphenols in DGLE with amino group of protein chains became lower. Notably, the C=O ester band at 1722 cm^−1^ observed in the AVP was attenuated in the surimi gel incorporated with AVP and the corresponding acetyl band shifted from 1260.92 cm^−1^ to 1245.70 cm^−1^. The attenuation and shift of the acetyl C=O band to a lower wavenumber implied their participation in hydrogen bonding with protein amide groups [6]. The band, observed at approximately 1052–1051 cm^−1^ across all treatment groups, showed only minor changes upon additive incorporation. The C-O stretching band of sugar moieties added as cryoprotectants to the surimi base was observed at 1052 cm^−1^ in the control gel, with a minor shift to 1051 cm^−1^ upon additive incorporation, suggesting minimal disruption of cryoprotectant-protein interactions by either AVP or DGLE [47]. The minimal interference observed indicated that the cryoprotectant had no negative effect on the myofibrillar protein interaction or aggregation in the presence of the incorporated additives.

### 2.9. Effect of AVP, DGLE and AVP + DGLE on Viscoelastic Properties

**Figure 3:**
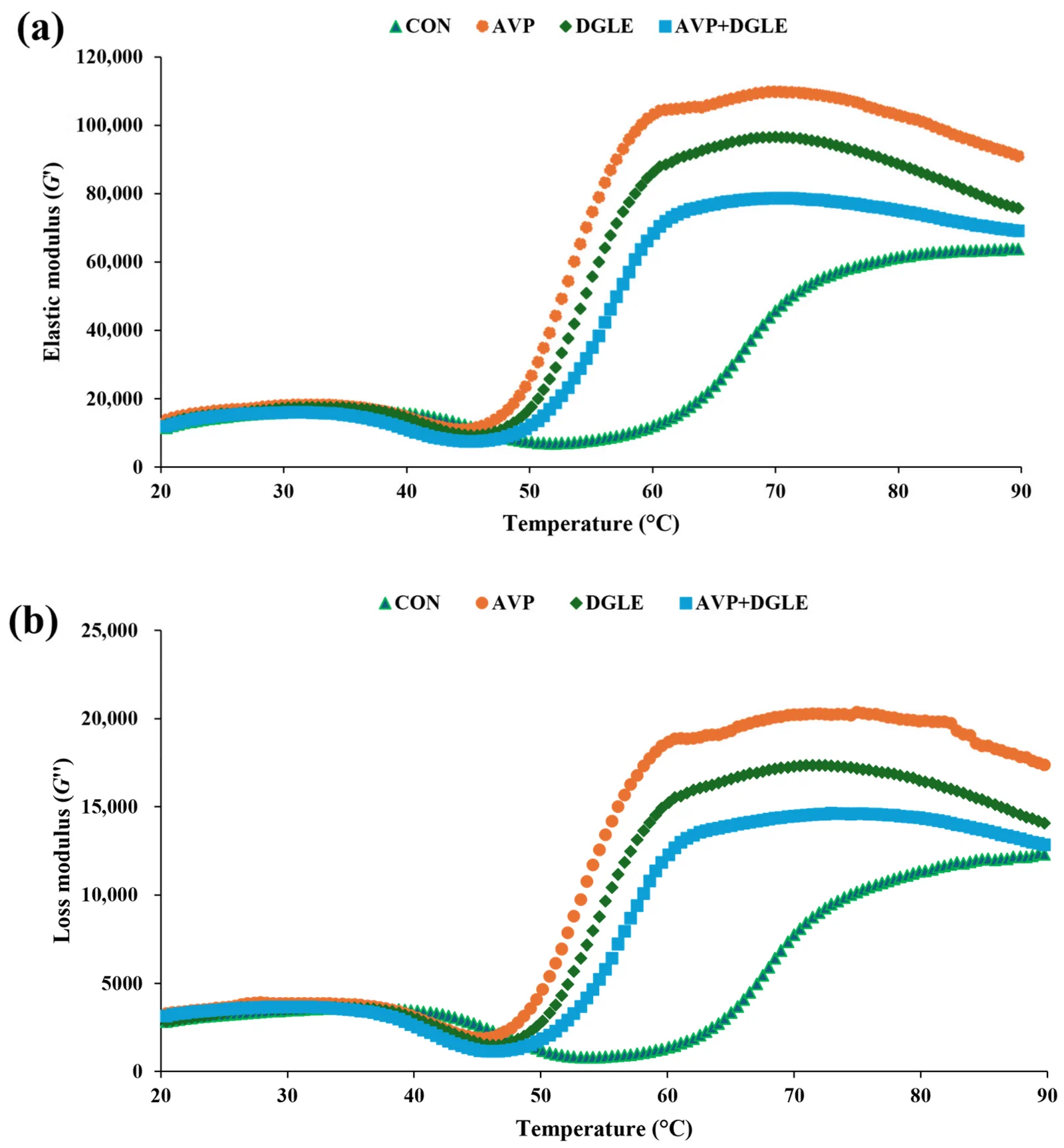
Elastic modulus (**a**) and loss modulus (**b**) of threadfin bream surimi paste without and with aloe vera powder, dechlorophyllized guava leaf extract and aloe vera powder and dechlorophyllized guava leaf extract combined over a temperature sweep of 20–90 °C. CON—surimi gel without any additives; AVP—surimi gel with 0.2% aloe vera powder; DGLE—surimi gel with 0.05% dechlorophyllized guava leaf extract; AVP + DGLE—surimi gel with 0.2% aloe vera powder and 0.05% dechlorophyllized guava leaf extract.

The elastic modulus (*G*′) and loss modulus (*G*″) determine the viscoelastic behavior of surimi gel, and the incorporation of additives substantially influenced these properties during heat-induced gelation [42]. Changes in *G*′ and *G*″ during the transition from sol to gel in threadfin bream surimi paste added with AVP (0.2%), DGLE (0.05%) and their combination (0.2% AVP + 0.05% DGLE) were compared with the control (no additives), and the results are presented in Figure 3.

In the control sample, an initial increase in *G*′ and *G*″ also exhibited initial rise during this phase. The increased viscous energy dissipation was observed during the setting stage (35–43 °C), attributed to protein aggregation of the S2 fragment in the heavy meromyosin region at low temperature [8]. The pronounced decline in *G*′ was observed at approximately 46.7 °C, with a corresponding increase in *G*″, indicating increased viscous flow. This was related with the activation of endogenous proteolytic enzymes primarily cysteine and serine proteinases, which are typically active at 50 °C in threadfin bream [47,48]. Proteolytic degradation of myofibrillar proteins increased protein fluidity, resulting in reduced *G*′ and the characteristic formation of modori gel [19]. In contrast, surimi pastes incorporated with AVP, DGLE and their combination exhibited slightly lower *G*′ and *G*″. than the control. The interaction between additives and proteins in surimi paste via weak bonds might decrease protein-protein interaction. The subsequent increase in *G*′ was drastic with rising temperature, which was in contrast to the behavior of control, which displayed lower increase. All samples had the lower *G*′ at 45–50 °C, indicating the gel weakening induced by indigenous protease in threadfin bream surimi. This gel-weakening phase coupled with accelerated sol-gel transition resembled the phenomena reported by Xiong et al. [49]. At temperature above 45 °C, thermal unfolding of proteins took place, facilitating the formation of covalent and non-covalent bonds as indicated by the increased *G*′. Gautam et al. [50] reported that TGase activation occurred mainly at temperature of 40–50 °C, thus promoting non-disulfide covalent bond formation among protein chains.

Higher *G*′ and *G*″ values were achieved in the additive treated samples. However, AVP and DGLE functioned differently in gelation of surimi paste. AVP, containing high polysaccharide content and hydrophilicity, might have formed additional physical hydrogel network through intermolecular cross-linking and reinforcing the protein matrix during gelation [51]. This was indicated by the highest *G*′ in sample added with AVP. DGLE, which was rich in polyphenolic compounds, might interact with myofibrillar proteins through covalent and non-covalent polyphenol-protein interactions. This might cause the aggregation between proteins, in which the bundle or coagulated proteins could not undergo aggregation properly. As a result, the lower *G*′ and *G*″ values were observed in comparison with that added with AVP. Yun et al. [31] found that tea polyphenols destabilized intramolecular forces within myosin in minced lamb meat, lowering thermal denaturation temperatures through alteration in protein chain conformation.

Upon further heating, the uncoiling and unfolding of light meromyosin chains promoted the formation of a thermo-irreversible three-dimensional gel network through extensive protein cross-linking, enhancing final gel strength [8]. AVP incorporated surimi achieved the highest *G*′ of 109,800 Pa, consistent with the TPA results, where AVP added sample exhibited the highest hardness. This finding aligned with observations by Lad and Murthy [52] who demonstrated that AVP exhibited solid-like viscoelastic behavior that progressively enhanced with increasing temperature. Surimi incorporated with DGLE attained peak *G*′ of 96,530 Pa, attributed to both non-covalent bonds and covalent cross-linking of proteins mediated by hydroxyl groups and quinone intermediates derived from polyphenol oxidation [37].

However, the combined AVP + DGLE treatment yielded an intermediate peak *G*′ of 78,680 Pa lower than either individual treatment, confirming less effective interaction between the two additives. However, all treatments still had the higher peak *G*′ than the control (63,310 Pa). The reduced gel strength in the combined system might be attributed to competitive interaction between polyphenolic compounds and polysaccharide for available binding sites on myosin or due to the formation of polysaccharide-polyphenol complexes that limit individual interactive capacity of each additive. Nevertheless, after reaching the peak of *G*′, a gradual decline in all samples was observed. This showed the similar trend to *G*″ values upon continued heating. This phenomenon was likely due to the disruption of weak hydrogen bonds within the gel network at elevated temperatures [6].

### 2.10. Effect of AVP, DGLE and Their Combination on Microscopic Images of Surimi Gel

**Figure 4:**
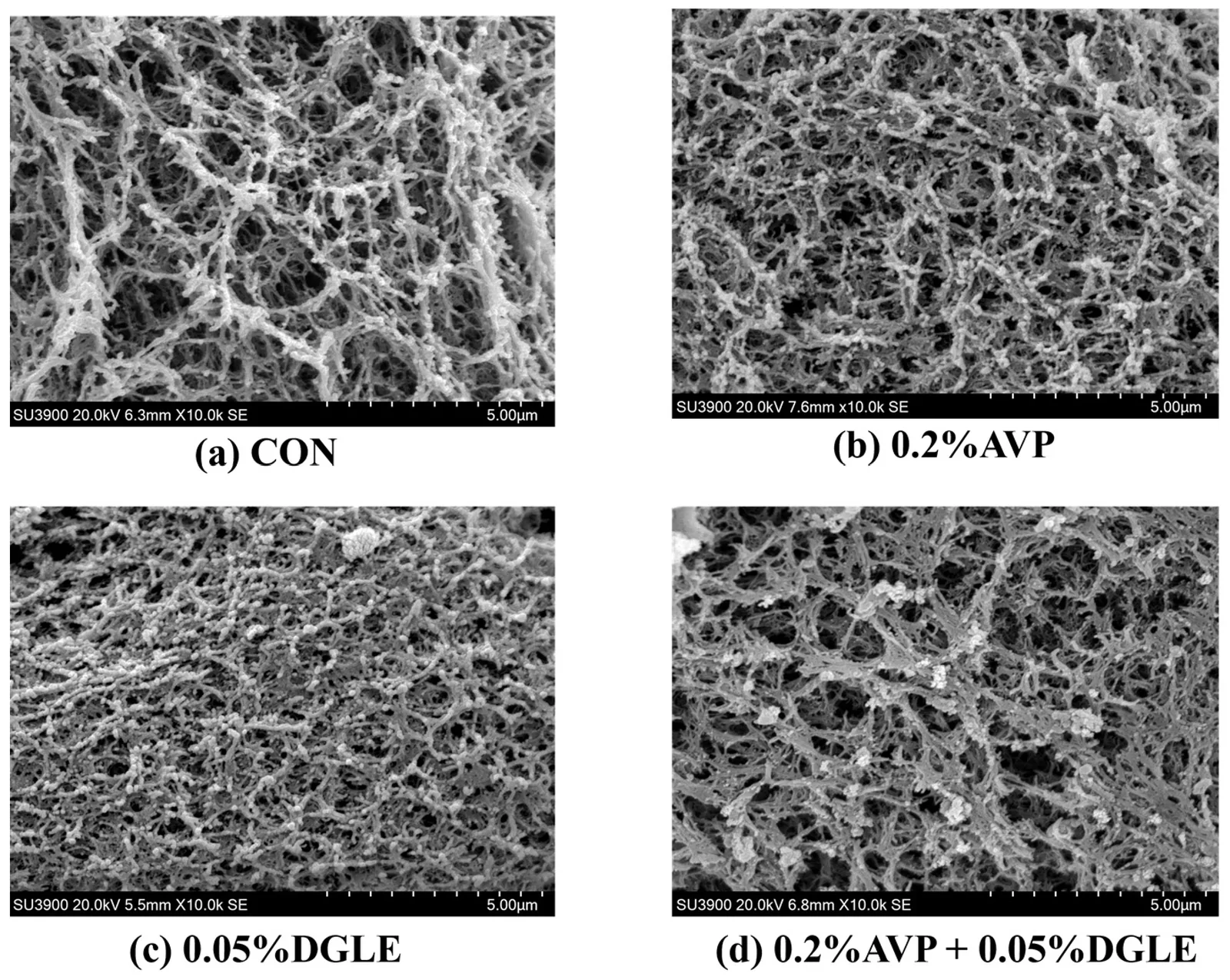
Scanning electron microscopic images of threadfin bream surimi gels; control without any additives (**a**); with 0.2% aloe vera powder (**b**); 0.05% dechlorophyllized guava leaf extract (**c**); combination of 0.2% aloe vera powder and 0.05% dechlorophyllized guava leaf extract (**d**); AVP—aloe vera powder; DGLE—dechlorophyllized guava leaf extract; (Magnification: 10,000×).

The microstructure images of the surimi gels added with 0.2% AVP, 0.05% DGLE and the combination of 0.2% AVP and 0.05% DGLE were examined by SEM in comparison to the control (no additives) as presented in Figure 4. The control sample had a coarser and irregular protein network characterized by larger pores arranged heterogeneously and loosely aggregated protein strands. This reflected the disorganized network formation, consistent with the threadfin bream surimi gel with no additives [23]. In addition, the coarser structure of the control was consistent with comparatively lower *G*′, hardness and water holding capacity observed in this study. Incorporation of AVP produced the most compact, uniform gel matrix as indicated by small pores distributed uniformly, consistent with the highest *G*′ and hardness due to the reinforced gel network. Besides, small pore size was related to the enhanced retention of water via secondary network formation by AVP [33], which was able to entrap water as shown by the low EMC of gel added with 0.2% AVP.

DGLE-incorporated gels established an improved network relative to the control and gel containing 0.2% AVP. This is consistent with enhanced pore uniformity and finer and denser protein aggregates. This was more likely mediated by higher protein cross-linking mediated by hydrogen bonds via the -OH group of polyphenols or quinone intermediates derived from polyphenol oxidation [11,37]. This structure supported the elevated *G*′ and cohesiveness as well as the lowest EMC.

The combined AVP + DGLE treatment yielded the gel with an intermediate microstructure characterized by greater network heterogeneity than either individual treatment. The larger and more irregularly distributed pores provided the morphological validation for the less effective interaction between polysaccharides and polyphenolic compounds in the combined ternary system. Collectively, these microstructural observations confirmed that AVP and DGLE individually enhanced the organization and density of the heat-induced surimi gel network. Nonetheless, their combination resulted in partial interference of these independent network-forming mechanisms, either by the formation of polysaccharide-polyphenol complexes or competition for myosin binding sites or dilution effect on myofibrillar proteins [23]. This might have resulted in a less ordered protein matrix, losing individual structural refinement achieved by either additive independently. Sample preparation steps for SEM, including fixation, dehydration, critical-point drying, and sputter-coating, might introduce minor artifacts such as shrinkage. Nonetheless, the consistent processing across all samples allowed for valid comparison of relative microstructural differences between treatments.

## 3. Conclusions

The incorporation of AVP and DGLE individually and in combination affected the quality of threadfin bream surimi gel differently, and the addition of DGLE rich in polyphenols (0.05%) to the surimi gel exhibited the highest B-F and D-F and low expressible moisture content. The addition of 0.2% AVP in surimi gel also showed improved textural properties and water-holding capacity, but the efficacy was lower than 0.05% DGLE. AVP- and DGLE-incorporated surimi gels exhibited the highest hardness and springiness, respectively. AVP incorporation positively impacted the whiteness of surimi gel, especially with increasing concentration, whereas DGLE reduced whiteness due to residual pigments. AVP-incorporated surimi possessed a higher final *G*′ than others, indicating that the entanglement of polysaccharide and protein chains during the heat-induced gelation process and sol-gel transition was promoted during thermal gelation. The combination of AVP and DGLE was less effective than the best individual treatment for gel-forming properties in the ternary system, where DGLE could not promote effective interactions with protein, likely due to the presence of AVP polysaccharides.

Collectively, the tested AVP-DGLE combinations did not outperform the best individual treatments, but true antagonism was not statistically established. Furthermore, the order of addition of AVP and DGLE, or their simultaneous addition, should be studied to elucidate the influence of the combination treatment. Thus, the incorporation of plant-derived additives alone could enhance the physicochemical, textural and rheological properties of surimi from threadfin bream more effectively than their combination. Further sensory evaluation is recommended to confirm consumer acceptability of the developed surimi gels, particularly regarding the incorporation of DGLE.

## 4. Materials and Methods

### 4.1. Raw Materials and Chemicals

Grade ‘A’ surimi blocks derived from threadfin bream (*Nemipterus* spp.) in a frozen state were procured commercially from Chaichareon Marine Company Ltd. (Pattani, Thailand) and stored at −20 °C until use. The surimi had a moisture content of 76.04% as determined by AOAC Method 928.08 [53]. Freeze-dried aloe vera powder free of aloin was procured from Yunnan Evergreen Biological Corporation (Yuxi, China). It had a loverose of 10.68%, aloin less than 0.1 ppm, and a moisture content of 3.01%. Guava leaf powder was prepared as described by Murugan et al. [54]. NaCl was procured from the Hat Yai local market. Chemical reagents of analytical grade were obtained from Sigma-Aldrich, Inc. (St. Louis, MO, USA), and all chemicals for electrophoresis were obtained from Bio-Rad Laboratories, Inc. (Richmond, CA, USA).

### 4.2. Preparation of Dechlorophyllized Guava Leaf Extract

Dechlorophylized guava leaf extract preparation was conducted following the method of Olatunde et al. [24] with slight changes. An ethanolic solution (70% *v*/*v*) was combined with guava leaf powder at a proportion of 1:15 (*w*/*v*), agitated at room temperature for 2 h with the speed of 300 rpm. Thereafter, ultrasonication (VC750, Sonics&Materials, Inc., Newtown, CT, USA) for 30 min with an amplitude of 70% in the pulse mode (50 s on/10 s off) was used. The mixture was immersed in an ice bath to prevent heat accumulation caused by ultrasonication. The resulting mixture was subsequently filtered (Whatman No. 4). To eliminate ethanol, the filtrate was kept under reduced pressure in a rotary evaporator (EV400H, LabTech Srl, Sorisole, Italy) at 40 °C, followed by nitrogen purging. To the resulting extract, distilled water (1:2 *v*/*v*) was added, mixed thoroughly, and kept for sedimentation for the duration of 24 h at 4 °C. The upper layer with no chlorophyll was carefully separated and centrifuged (Model Himac CR22N, Hitachi Koki Co., Ltd., Hitachinaka, Japan) (10,000× *g*, 4 °C, 30 min). The supernatant obtained was lyophilized to yield 12.76% ± 0.22 dry powder, which was referred to as ‘DGLE’. The powder was packaged in a zip-lock bag and stored at −20 °C.

### 4.3. Characterization of DGLE

#### 4.3.1. Total Phenolic Content (TPC) of DGLE

TPC present in DGLE was quantified using the Folin-Ciocalteu (FC) method [55]. Concisely, 10 µL of DGLE solution was combined with 75 µL of 10% FC reagent, followed by keeping the mixture at room temperature (RT) in the dark for 5 min. Thereafter, 6% sodium carbonate solution (75 µL) was added, and the mixture was left to react at room temperature in the dark for 1 h. Gallic acid standards (0–500 mg/L) were used to construct a calibration curve. Absorbance was measured using a microplate reader (FLUOstar^®^, Omega, Ortenberg, Germany) at 765 nm. TPC was reported as mg gallic acid equivalents (GAE)/g dry weight of freeze-dried DGLE powder.

#### 4.3.2. Total Flavonoid Content (TFC) of DGLE

TFC in DGLE was quantified via aluminum chloride colorimetric assay [56]. Concisely, the sample (20 µL) was combined with 80 µL of distilled water and 6 µL of 5% (*w*/*v*) sodium nitrite solution. After 5 min of reaction, 6 µL of 10% (*w*/*v*) aluminum chloride solution was added. After 6 min, 40 µL of NaOH (1 M) was added, and distilled water was used to adjust the volume to 200 µL. Absorbance was read at 510 nm in a microplate reader (FLUOstar^®^, Omega, Germany) in triplicate. TFC was calculated using a quercetin standard curve (0–300 mg/L) and expressed as mg quercetin equivalent (QE)/g dry weight of freeze-dried DGLE powder.

#### 4.3.3. Profiling of Phenolic Compounds in DGLE

Phenolic compounds present in DGLE were analyzed using LC-MS/MS, X500 QTOF (SCIEX, Framingham, MA, USA) [57]. The extract was prepared at a concentration of 1000 ppm in methanol, and 5 µL was injected per run. Electrospray ionization (ESI) was employed as the ionization source, in both positive and negative ionization modes. A C8 column maintained at 30 °C was utilized for chromatographic separation using a binary mobile phase system consisting of 0.1% aqueous formic acid and acetonitrile delivered at a flow rate of 1 mL/min under gradient elution conditions. SCIEX OS 2.0.0 software was used to acquire and process the data using the NIST tandem mass spectral library.

### 4.4. Preparation of Surimi Gel

#### Preparation of Surimi Gel Added with AVP, DGLE and AVP + DGLE

Frozen surimi was allowed to thaw overnight at 4 °C to reach a core temperature of 2 °C. The method of Singh et al. [18] was adopted for surimi gel preparation. Surimi (100 g) was chopped and blended in a food processor (MK-5087M, Panasonic, Shah Alam, Malaysia) for 1 min, after which 2.5% (*w*/*w*) NaCl was added. The blending was conducted for 1 min. Chilled distilled water was then added to adjust the moisture content to 80%, and further mixing was done for 1 min. AVP and DGLE at various concentrations of 0%, 0.05%, 0.1%, 0.2%, 0.3%, 0.4% and 0.5% (*w*/*w*) based on the total weight of the surimi were incorporated and blended for 2 min. A sample without addition of either AVP or DGLE was used as the control. All preparation was done in a walk-in cold room, keeping the surimi paste temperature below 10 °C. The surimi paste was then filled into polyvinylidene chloride (PVDC) casings (internal diameter: 2.5 cm) and sealed tightly at both ends. Gelation was carried out in a water bath using a two-step heating method: setting at 40 °C for 30 min, followed by cooking at 90 °C for 20 min. The gels were transferred to an ice-water bath and kept for 15 min. Finally, the resulting gel samples were then stored at 4 °C for 18 h.

To study the impact of the combination of AVP and DGLE, AVP at the level of 0.2% was used. DGLE at different concentrations (0%, 0.05%, 0.1% and 0.2% *w*/*w*) was added after the addition of AVP at the designated level. DGLE at higher individual concentrations (0.3–0.5%) were excluded in the combination study to prevent excessive polyphenol-polyphenol interaction or polyphenol-induced protein destabilization. The mixture was blended for another 2 min. The gel without and with AVP or DGLE at the optimal level showing the highest textural properties was considered as the control.

### 4.5. Analyses of Surimi Gel Properties

#### 4.5.1. Breaking Force and Deformation

The maximum force required to rupture the gel and the distance of the point where the rupture of the gel occurred was measured as breaking force (g) and deformation (mm), respectively, using a TA-XT2 texture analyzer (Stable MicroSystems, Surrey, UK) with a 50 kg load cell and a 5 mm spherical probe (P/5S) [19]. Cylindrical-shaped surimi gels having 2.5 cm in diameter and height were cut and left to temper at RT for 30 min before testing. Penetration testing parameters included a pre-test speed of 1.0 mm/s, test speed of 1.1 mm/s, and post-test speed of 10.0 mm/s. The probe travelled a distance of 15 mm with a trigger force set at 10 g.

#### 4.5.2. Texture Profile Analysis (TPA)

TPA was conducted using a TA-XT2 texture analyzer (Stable MicroSystems, Surrey, UK) equipped with a 50 kg load cell and a 40 mm cylindrical aluminum probe (P/40). The sample was positioned for compression of two cycles with a time interval of 5 s and compressed to 60% of its initial height with a pre-test speed of 1 mm/s, test speed and post-test speed of 5 mm/s and trigger force of 10 g.

#### 4.5.3. Expressible Moisture Content (EMC)

EMC was determined to evaluate the water-holding capacity of surimi gels [6]. A surimi gel slice (5 mm thickness) was positioned between two layers of Whatman No. 4 filter paper on top and three layers at the bottom. Followed by compression for 2 min with a constant 5 kg standard weight, samples were weighed before (*X*) and after (*Y*) compression. The percentage of EMC was assessed as the proportion of water released upon compression relative to the initial sample weight (Equation (1)).

(1) $$EMC\left(\%\right)=\left[\frac{X-Y}{X}\right]\;100$$

#### 4.5.4. Whiteness

Whiteness of the surimi gels was determined using a colorimeter (ColorFlex, Hunter Lab, Reston, VA, USA) with a 45°/0° geometry. Instrument calibration was performed using black-and-white standard tiles in succession. Measurements were taken under Illuminant D65 with a 10° standard observer. Color was compared with the white standard tile (L* = 94.89, a* = −1.17, b* = 0.88) and was recorded as CIE L*, a* and b* values. Whiteness of the surimi gel was assessed according to Murugesan et al. [58] with Equation (2).

(2) $$Whiteness=100-[{\left(100-L^{*}\right)}^{2}+a^{*2}+b^{*2}{]}^{1/2}$$

#### 4.5.5. Sodium Dodecyl Sulfate-Polyacrylamide Gel Electrophoresis (SDS-PAGE)

SDS-PAGE was performed to evaluate the protein pattern of surimi gel samples according to Petcharat and Benjakul [19] with minor modifcation. Roughly 3 g of minced surimi gel sample was combined with 27 mL of preheated (85 °C) 5% SDS solution, homogenized (11,000 rpm, 2 min), incubated (85 °C for 1 h), and finally centrifuged (3500× *g*, 20 min) at RT. The supernatant was separated and quantified for protein content via the Biuret assay. Samples were mixed with reducing sample buffer, heated at 90 °C for 3 min, followed by loading the mixture containing 30 µg protein into each well of the polyacrylamide gel consisting of a 7.5% resolving gel and a 4% stacking gel, prepared according to the Laemmli method [59]. After electrophoresis, the gel was stained with Coomassie Brilliant Blue R-250 solution, and then destained. A long-range molecular weight protein standard (ColorBurst, Sigma-Aldrich) was used. Rf of proteins in standard and those of gel samples were computed and used for estimation of molecular weight.

#### 4.5.6. Fourier Transform Infrared Spectroscopic (FT-IR) Analysis

The secondary protein structure of surimi gel samples (freeze-dried) was analyzed by FTIR spectroscopy with a Bruker INVENIO S FTIR spectrometer (BrukerCo., Ettlingen, Germany). Prior to analysis, finely ground samples were blended with potassium bromide (KBr) at a ratio of 1:10 (*w*/*w*, sample: KBr) and compressed with a hydraulic press into transparent pellets of 7 mm. Spectra were collected across a spectral range between 4000–400 cm^−1^ by averaging 32 scans at a 4 cm^−1^ resolution. The amide I band region (1600–1700 cm^−1^) was used to evaluate changes in protein secondary structure by spectral deconvolution using PeakFit v4.12 software [43]. After linear baseline subtraction, component bands within the amide I region were identified in the second-derivative spectrum (Savitzky–Golay smoothing, software-selected level) and fitted with a Gaussian peak-shape function using automatically estimated initial amplitudes. No manual constraints were applied to peak position, width, or amplitude, and all fits achieved R^2^~0.99. Bands at 1618–1640, 1640–1650, 1650–1660 and 1660–1690 cm^−1^ were assigned to β-sheet, random coil, α-helix and β-turn structures, respectively [3]. The relative content of each secondary structure was calculated as the percentage of its fitted peak area relative to the total amide I band area.

#### 4.5.7. Dynamic Rheological Measurement

Rheological analysis followed the protocol described by Benjakul et al. [60]. The viscoelastic properties of surimi paste were determined using a rheometer (HAAKE Rheostress1, Thermo Fisher Scientific, Karlsruhe, Germany) with a 35 mm, 2° cone-and-plate geometry. The paste was loaded onto the plate with the measurement gap at 1 mm. The excess at the edge of the geometry was trimmed prior to measurement. The temperature sweep was then immediately started without an equilibration hold period. A temperature sweep at a steady heating rate of 1 °C/min from 20 to 90 °C was conducted to monitor the elastic (*G*′) and loss (*G*″) moduli at a fixed frequency of 1 Hz and a strain of 1%. A preliminary oscillatory amplitude sweep confirmed these parameters fell within the LVR. Each sample was performed in triplicate. A solvent trap was employed throughout the measurement to minimize evaporation from the tested samples.

#### 4.5.8. Microstructural Analysis

Microstructural characterization of surimi gel matrix was conducted using a scanning electron microscope (Hitachi, SU3900, Tokyo, Japan). Samples were prepared according to Petcharat and Benjakul [19] with minor modification. Samples of 2–3 mm thickness were fixed with 2.5% glutaraldehyde solution prepared in 0.2 M phosphate buffer (pH 7.2) for 3 h at 4 °C. Then, gels were rinsed with distilled water and serially dehydrated for 15 min each in ethanol (25, 50, 70, 80, 90 and 100%, *v*/*v*). Finally, the dried samples were obtained via critical point drying with liquid CO_2_ and placed on a bronze stub. Gold sputter-coated samples were prepared to render the samples electrically conductive prior to imaging.

### 4.6. Statistical Analysis

A completely randomized design (CRD) was used for the entire experiment. All experiments and analyses were performed in triplicate, and the data were expressed as mean ± standard deviation (SD). One-way analysis of variance (ANOVA) was employed for overall treatment differences. Mean comparisons were subsequently performed using Duncan’s multiple range. All calculations were performed using SPSS (version 22; IBM Corp., Armonk, NY, USA), with a significance level of *p* < 0.05.

## Figures and Tables

**Table 1:** Phenolic compounds in the dechlorophyllized guava leaf extract as determined by LCMS/MS QTOF.

Compound	Mode	R_t_ (min)	*m*/*z*	MW	Area
(−)-Catechin	negative	7.50	289.07	290.07	3.987 × 10^6^
Epicatechin	negative	7.49	579.15	290.07	1.125 × 10^6^
Hyperoside	negative	7.54	463.05	464.06	2.872 × 10^6^
(−)-Epigallocatechin Gallate	negative	8.46	457.17	458.08	1.257 × 10^6^
Ellagic acid	negative	9.45	300.995	302.00	1.690 × 10^7^
(−)-Epicatechin gallate	negative	10.03	441.08	442.09	3.065 × 10^6^
Kaempferol	negative	13.01	285.04	286.04	4.201 × 10^6^
(−)-Gallocatechin	positive	5.72	307.08	306.07	8.993 × 10^6^
(+)-Catechin	positive	7.49	291.08	290.07	1.427 × 10^7^
Quercetin	positive	9.59	303.05	302.04	8.189 × 10^6^
Guaiaverin	positive	10.26	435.09	434.08	7.267 × 10^6^
Kaempferol	positive	13.01	287.05	286.04	5.606 × 10^5^

**Table 2:** Breaking force and deformation of threadfin bream surimi gel incorporated with AVP, DGLE, and their combination at different levels.

Additive	Concentration (%)	Breaking Force (g)	Deformation (mm)	Expressible Moisture Content (%)
Control	-	295.70 ± 1.94 ^g^	7.50 ± 0.30 ^b^	4.96 ± 0.22 ^def^
AVP	0.05	281.48 ± 11.02 ^fg^	7.15 ± 0.07 ^f^	4.75 ± 0.07 ^fg^
	0.1	298.10 ± 19.09 ^f^	7.26 ± 0.11 ^ef^	4.67 ± 0.26 ^gh^
	0.2	395.11 ± 7.15 ^b^	9.3 ± 0.30 ^a^	4.47 ± 0.41 ^gh^
	0.3	358.41 ± 3.89 ^cd^	8.26 ± 0.05 ^b^	5.68 ± 0.25 ^de^
	0.4	343.82 ± 8.45 ^d^	8.23 ± 0.15 ^b^	6.59 ± 0.38 ^bc^
	0.5	343.66 ± 2.24 ^d^	7.9 ± 0.17 ^c^	7.51 ± 0.27 ^ab^
DGLE	0.05	401.81 ± 5.21 ^a^	9.06 ± 0.37 ^a^	4.37 ± 0.31 ^gh^
	0.1	322.66 ± 12.88 ^e^	7.73 ± 0.11 ^cd^	4.70 ± 0.01 ^gh^
	0.2	295.20 ± 7.46 ^g^	7.46 ± 0.05 ^de^	5.00 ± 0.25 ^e^
	0.3	291.94 ± 3.89 ^f^	7.16 ± 00.11 ^f^	6.07 ± 0.70 ^de^
	0.4	265.40 ± 0.74 ^gh^	6.95 ± 0.05 ^g^	7.28 ± 0.16 ^ab^
	0.5	258.49 ± 6.27 ^hi^	6.88 ± 0.07 ^g^	7.45 ± 0.20 ^a^
AVP + DGLE	0.2 + 0.05	382.75 ± 1.06 ^b^	8.43 ± 0.05 ^b^	4.57 ± 0.10 ^gh^
	0.2 + 0.1	375.94 ± 3.59 ^b^	8.23 ± 0.20 ^b^	4.84 ± 0.27 ^fg^
	0.2 + 0.2	367.50 ± 50 ^bc^	7.65 ± 0.07 ^cd^	5.43 ± 0.28 ^def^

Values are expressed as mean ± standard deviation (*n* = 3). Different lowercase superscripts within the same column indicate significant differences (*p* < 0.05). Control—surimi gel without additives; AVP—aloe vera powder; DGLE—dechlorophyllized guava leaf extract; AVP + DGLE—aloe vera powder in combination with dechlorophyllized guava leaf extract.

**Table 3:** Texture profile of threadfin bream surimi gel incorporated with AVP, DGLE and their combination at different levels.

Samples	Hardness (g)	Springiness	Cohesiveness	Gumminess (g)	Chewiness (g)
Control	9545.37 ± 17.7 ^e^	0.87 ± 0.00 ^f^	0.71 ± 0.01 ^b^	7246.09 ± 165.2 ^d^	7257.39 ± 218.38 ^bc^
0.05% AVP	9452.16 ± 56.28 ^ef^	0.88 ± 0.00 ^e^	0.72 ± 0.01 ^b^	7385.23 ± 122.32 ^d^	5780.08 ± 116.38 ^e^
0.1% AVP	9947.61 ± 20.05 ^d^	0.87 ± 0.00 ^f^	0.71 ± 0.01 ^b^	7039.71 ± 292.82 ^d^	6400.27 ± 292.82 ^d^
0.2% AVP	11,436.43 ± 105.5 ^a^	0.91 ± 0.00 ^b^	0.75 ± 0.00 ^a^	8669.43 ± 82.68 ^a^	7902.89 ± 96.21 ^a^
0.3% AVP	8116.32 ± 77.76 ^i^	0.88 ± 0.00 ^e^	0.47 ± 0.01 ^d^	3511.55 ± 165.20 ^g^	3167.51 ± 102.06 ^g^
0.4% AVP	7538.77 ± 2.16 ^j^	0.87 ± 0.01 ^f^	0.48 ± 0.02 ^d^	3230.24 ± 128.25 ^g^	3101.56 ± 118.68 ^g^
0.5% AVP	7215.18 ± 146.76 ^k^	0.86 ± 0.00 ^g^	0.37 ± 0.01 ^f^	2903.21 ± 77.59 ^h^	2541.00 ± 131.92 ^h^
0.05% DGLE	11,115.83 ± 28.26 ^b^	0.94 ± 0.00 ^a^	0.76 ± 0.00 ^a^	8526.54 ± 80.01 ^ab^	7870.35 ± 100.24 ^a^
0.1% DGLE	9485.93 ± 176.95 ^ef^	0.88 ± 0.00 ^e^	0.71 ± 0.00 ^b^	6630.18 ± 180.22 ^e^	5862.23 ± 148.85 ^e^
0.2% DGLE	9154.64 ± 142.21 ^gh^	0.89 ± 0.00 ^d^	0.71 ± 0.00 ^b^	6511.91 ± 265.94 ^e^	5798.37 ± 251.38 ^e^
0.3% DGLE	8969.70 ± 26.18 ^h^	0.88 ± 0.00 ^e^	0.69 ± 0.02 ^b^	6390.33 ± 148.85 ^e^	5658.68 ± 109.54 ^ef^
0.4% DGLE	7385.17 ± 129.85 ^jk^	0.87 ± 0.00 ^f^	0.44 ± 0.00 ^e^	3276.75 ± 36.47 ^g^	2874.18 ± 72.93 ^g^
0.5% DGLE	6133.17 ± 96.76 ^l^	0.87 ± 0.01 ^f^	0.35 ± 0.03 ^f^	2643.23 ± 25.21 ^h^	2257.93 ± 56.21 ^i^
0.2% AVP + 0.05% DGLE	10,940.80 ± 19.10 ^bc^	0.90 ± 0.00 ^c^	0.75 ± 0.00 ^a^	8278.98 ± 33.59 ^bc^	7548.65 ± 71.21 ^b^
0.2% AVP + 0.1% DGLE	10,867.83 ± 127.16 ^c^	0.90 ± 0.00 ^c^	0.74 ± 0.00 ^a^	8127.25 ± 157.53 ^c^	7382.76 ± 97.83 ^b^
0.2% AVP + 0.2% DGLE	9590.96 ± 136.60 ^e^	0.89 ± 0.00 ^d^	0.64 ± 0.01 ^c^	5872.39 ± 6.22 ^f^	5374.13 ± 6.22 ^f^

Values are expressed as mean ± standard deviation (*n* = 3). Different lowercase superscripts within the same column indicate significant differences (*p* < 0.05). Control—surimi gel without additives; AVP—surimi gel incorporated with aloe vera powder; DGLE—surimi gel incorporated with dechlorophyllized guava leaf extract with 0.05, 0.1, 0.2, 0.3, 0.4 and 0.5% concentration; 0.2% AVP + 0.05% DGLE—surimi gel with 0.2% aloe vera powder and 0.05% of dechlorophyllized guava leaf extract; 0.2% AVP + 0.1% DGLE: surimi gel with 0.2% aloe vera powder and 0.1% of dechlorophyllized guava leaf extract; 0.2% AVP + 0.2% DGLE: surimi gel with 0.2% aloe vera powder and 0.2% of dechlorophyllized guava leaf extract.

**Table 4:** L*, a*, b* and whiteness of threadfin bream surimi gel incorporated with AVP, DGLE and their combination at different levels.

Sample	L*	a*	b*	Whiteness
Control	77.19 ± 0.20 ^d^	0.03 ± 0.00 ^j^	13.19 ± 0.07 ^e^	73.69 ± 0.12 ^c^
0.05% AVP	77.21 ± 0.22 ^d^	0.04 ± 0.00 ^j^	13.15 ± 0.02 ^e^	73.66 ± 0.25 ^c^
0.1% AVP	77.30 ± 0.28 ^d^	0.01 ± 0.03 ^j^	13.11 ± 0.06 ^e^	73.78 ± 0.21 ^c^
0.2% AVP	77.22 ± 0.28 ^d^	0.05 ± 0.01 ^j^	13.07 ± 0.12 ^e^	73.73 ± 0.24 ^c^
0.3% AVP	77.68 ± 0.27 ^c^	0.12 ± 0.08 ^i^	12.9 ± 0.20 ^e^	74.20 ± 0.14 ^b^
0.4% AVP	78.05 ± 0.02 ^b^	0.14 ± 0.01 ^i^	12.9 ± 0.05 ^e^	74.54 ± 0.00 ^b^
0.5% AVP	78.67 ± 0.14 ^a^	0.26 ± 0.07 ^gh^	12.4 ± 0.05 ^f^	75.31 ± 0.13 ^a^
0.05% DGLE	73.54 ± 0.01 ^e^	2.43 ± 0.01 ^f^	11.16 ± 0.04 ^d^	71.18 ± 0.02 ^d^
0.1% DGLE	73.04 ± 0.03 ^e^	2.99 ± 0.03 ^d^	11.70 ± 0.09 ^h^	70.45 ± 0.04 ^e^
0.2% DGLE	72.35 ± 0.12 ^f^	3.26 ± 0.05 ^c^	13.06 ± 0.04 ^e^	69.24 ± 0.10 ^f^
0.3% DGLE	71.71 ± 0.06 ^g^	3.77 ± 0.05 ^b^	15.05 ± 0.07 ^c^	67.73 ± 0.06 ^g^
0.4% DGLE	71.26 ± 0.21 ^g^	4.18 ± 0.03 ^a^	16.45 ± 0.07 ^b^	66.61 ± 0.14 ^h^
0.5% DGLE	70.18 ± 0.23 ^h^	4.19 ± 0.03 ^a^	16.66 ± 0.06 ^a^	65.58 ± 0.04 ^i^
0.2% AVP + 0.05% DGLE	72.63 ± 0.09 ^f^	2.40 ± 0.06 ^f^	11.08 ± 0.12 ^i^	71.38 ± 0.05 ^d^
0.2% AVP + 0.1% DGLE	72.15 ± 0.14 ^f^	2.72 ± 0.06 ^e^	11.99 ± 0.05 ^g^	70.55 ± 0.12 ^e^
0.2% AVP + 0.2% DGLE	71.72 ± 0.23 ^g^	3.18 ± 0.10 ^c^	13.41 ± 0.13 ^d^	69.54 ± 0.25 ^f^

Values are expressed as mean ± standard deviation (*n* = 3). Different lowercase superscripts within the same column indicate significant differences (*p* < 0.05). Control—surimi gel without additives; AVP—surimi gel incorporated with aloe vera powder; DGLE—surimi gel incorporated with dechlorophyllized guava leaf extract with 0.05, 0.1, 0.2, 0.3, 0.4 and 0.5% concentration; 0.2% AVP + 0.05% DGLE: surimi gel with 0.2% aloe vera powder and 0.05% of dechlorophyllized guava leaf extract; 0.2% AVP + 0.1% DGLE—surimi gel with 0.2% aloe vera powder and 0.1% of dechlorophyllized guava leaf extract; 0.2% AVP + 0.2% DGLE: surimi gel with 0.2% aloe vera powder and 0.2% of dechlorophyllized guava leaf extract; L*—lightness; a*—redness/greenness; b*—yellowness/blueness.

## Data Availability

The data used to support the findings of this study can be made available by the corresponding author upon request.

## References

[1] FAO (2024). The State of World Fisheries and Aquaculture 2024. Blue Transformation in Action.

[2] Bremenkamp I., Sousa-Gallagher M.J. (2024). Design and development of an edible coating for a ready-to-eat fish product. Polymers.

[3] Chen J., Hu Y., Gao P., Jiang Q., Yu P., Yang F., Pan M., Zhou X., Xia W. (2024). Effect of κ-carrageenan on the physicochemical and structural characteristics of ready-to-eat Antarctic krill surimi gel. Int. J. Food Sci. Technol..

[4] Vidal-Giraud B., Chateau D. (2007). World Surimi Market.

[5] Tadpitchayangkoon P., Park J.W., Yongsawatdigul J. (2012). Gelation characteristics of tropical surimi under water bath and ohmic heating. LWT Food Sci. Technol..

[6] Sompongse W., Hongviangjan W., Sakamut P. (2024). Interaction of polysaccharides on gel characteristics and protein microstructure of threadfin bream surimi gel. Int. J. Food Sci. Technol..

[7] Yi S., Li Q., Qiao C., Zhang C., Wang W., Xu Y., Mi H., Li X., Li J. (2020). Myofibrillar protein conformation enhance gel properties of mixed surimi gels with *Nemipterus virgatus* and *Hypophthalmichthys molitrix*. Food Hydrocoll..

[8] Zhao X., Mei T., Cui B. (2024). Effect and mechanism of different exogenous biomolecules on the thermal-induced gel properties of surimi: A review. J. Food Sci..

[9] Han G., Li Y. (2024). A review of inhibition mechanisms of surimi protein hydrolysis by different exogenous additives and their application in improving surimi gel quality. Food Chem..

[10] Walayat N., Blanch M., Moreno H.M. (2025). Surimi and low-salt surimi gelation: Key components to enhance the physicochemical properties of gels. Gels.

[11] Singh A., Mehta S., Patil U., Nuthong P., Benjakul S. (2023). Hyperoside improves the textural and rheological properties of threadfin bream surimi gel. Int. J. Food Sci. Technol..

[12] Sharma S., Majumdar R.K., Mehta N.K. (2022). Gelling properties and microstructure of the silver carp surimi treated with pomegranate (*Punica granatum* L.) peel extract. J. Food Sci. Technol..

[13] Huynh H.D., Nargotra P., Wang H.-M.D., Shieh C.-J., Liu Y.-C., Kuo C.-H. (2025). Bioactive compounds from guava leaves (*Psidium guajava* L.): Characterization, biological activity, synergistic effects, and technological applications. Molecules.

[14] Wani K.M., Uppaluri R.V.S. (2022). Efficacy of ultrasound-assisted extraction of bioactive constituents from *Psidium guajava* leaves. Appl. Food Res..

[15] Díaz-de-Cerio E., Gómez-Caravaca A.M., Verardo V., Fernández-Gutiérrez A., Segura-Carretero A. (2016). Determination of guava (*Psidium guajava* L.) leaf phenolic compounds using HPLC-DAD-QTOF-MS. J. Funct. Foods.

[16] Zheng M., Liu X., Chuai P., Jiang Z., Zhu Y., Zhang B., Ni H., Li Q. (2021). Effects of crude fucoidan on physicochemical properties, antioxidation and bacteriostasis of surimi products. Food Control.

[17] Zhuang X., Wang L., Jiang X., Chen Y., Zhou G. (2020). The effects of three polysaccharides on the gelation properties of myofibrillar protein: Phase behaviour and moisture stability. Meat Sci..

[18] Pati B.K., Priyadarshini M.B., Mehta N.K., Xavier K.A.M., Patel A.B., Upadhyay A.D., Singh S.K., Singh N.S., Vaishnav A., Gaurav K. (2025). Influence of snow crab shell derived chitooligosaccharide on the physicochemical, gelation and structural attributes of pangasius surimi. J. Agric. Food Res..

[19] Petcharat T., Benjakul S. (2018). Effect of gellan incorporation on gel properties of bigeye snapper surimi. Food Hydrocoll..

[20] Zhang T., Xue Y., Li Z., Wang Y., Xue C. (2015). Effects of deacetylation of konjac glucomannan on Alaska Pollock surimi gels subjected to high-temperature (120 °C) treatment. Food Hydrocoll..

[21] Hamman J.H. (2008). Composition and applications of aloe vera leaf gel. Molecules.

[22] Kim D.-H., Lee H., Yang H.-W., Jeon Y.-J. (2024). Immuno-enhancing effects of surimi products including functional ingredients in vitro and in vivo zebrafish model. Fish. Aquat. Sci..

[23] Xue H., Feng J., Tang Y., Wang X., Tang J., Cai X., Zhong H. (2024). Research progress on the interaction of the polyphenol–protein–polysaccharide ternary systems. Chem. Biol. Technol. Agric..

[24] Olatunde O.O., Tan S.L.D., Shiekh K.A., Benjakul S., Nirmal N.P. (2021). Ethanolic guava leaf extracts with different chlorophyll removal processes: Anti-melanosis, antibacterial properties and the impact on qualities of Pacific white shrimp during refrigerated storage. Food Chem..

[25] Singh S., Chidrawar V.R., Hermawan D., Nwabor O.F., Olatunde O.O., Jayeoye T.J., Samee W., Ontong J.C., Chittasupho C. (2023). Solvent-assisted dechlorophyllization of *Psidium guajava* leaf extract: Effects on the polyphenol content, cytocompatibility, antibacterial, anti-inflammatory, and anticancer activities. S. Afr. J. Bot..

[26] Lorena C., Ressaissi A., Serralheiro M.L. (2022). Bioactives from Psidium guajava leaf decoction: LC-HRMS-MS-QTOF identification, bioactivities and bioavailability evaluation. Food Chem. Adv..

[27] Chowdhury T.A., Islam M.M., Barmon J., Rana G.M.M., Uddin M.J., Ghos B.C., Karmakar A., Dey S.S., Rashid M.B., Chaki B.M. (2026). Comparative GC–MS and FTIR profiling with assessment of antioxidant and antimicrobial potential in guava, bay and lemon leaf extracts. Sustain. Chem. Environ..

[28] Zhu Y., Zhang J., Chen F., Hu X., Xu D., Cao Y. (2021). Epimerization and hydrolysis of catechins under ultrasonic treatment. Int. J. Food Sci. Technol..

[29] March R.E., Miao X.S. (2004). A fragmentation study of kaempferol using electrospray quadrupole time-of-flight mass spectrometry at high mass resolution. Int. J. Mass Spectrom..

[30] Chuang S.-Y., Lin Y.-K., Lin C.-F., Wang P.-W., Chen E.-L., Fang J.-Y. (2017). Elucidating the skin delivery of aglycone and glycoside flavonoids: How the structures affect cutaneous absorption. Nutrients.

[31] Yun X., Yang G., Li L., Wu Y., Yang X., Gao A. (2025). The effects of tea polyphenols on the emulsifying and gelling properties of minced lamb after repeated freeze–thaw cycles. Foods.

[32] Xie D., Tang Y., Dong G. (2024). Various factors affecting the gel properties of surimi: A review. J. Texture Stud..

[33] Yuan L., Yu J., Mu J., Shi T., Sun Q., Jin W., Gao R. (2019). Effects of deacetylation of konjac glucomannan on the physico-chemical properties of surimi gels from silver carp (*Hypophthalmichthys molitrix*). RSC Adv..

[34] Minjares-Fuentes R., Femenia A., Comas-Serra F., Rosselló C., Rodríguez-González V.M., González-Laredo R.F., Gallegos-Infante J.A., Medina-Torres L. (2016). Effect of different drying procedures on physicochemical properties and flow behavior of Aloe vera (*Aloe barbadensis* Miller) gel. LWT.

[35] Yan W., Yin T., Xiong S., You J., Hu Y., Huang Q. (2021). Gelling properties of silver carp surimi incorporated with konjac glucomannan: Effects of deacetylation degree. Int. J. Biol. Macromol..

[36] Balange A., Benjakul S. (2009). Enhancement of gel strength of bigeye snapper (*Priacanthus tayenus*) surimi using oxidized phenolic compounds. Food Chem..

[37] Vate N.K., Benjakul S. (2016). Effect of the mixtures of squid ink tyrosinase and tannic acid on properties of sardine surimi gel. J. Food Sci. Technol..

[38] Zhou F., Jiang W., Tian H., Wang L., Zhu J., Luo W., Liang J., Xiang L., Cai X., Wang S. (2024). Influence of EGCG (epigallocatechin gallate) on physicochemical–rheological properties of surimi gel and mechanism based on molecular docking. Foods.

[39] Duan L., Huang Y., Liu W., Zhang J., Wang Y., Zhang Y., Liang W., Pang J., Wu C. (2026). The effect of polygonatum polysaccharides on the gel properties and protein conformations of pacific white shrimp surimi gel. Food Biophys..

[40] Lv Y.-Y., Zhang X.-H., Zhou J.-N., Wang Y.-R., Zhang Z.-J., Yan J.-N., Lai B., Wang C., Wu H.-T. (2026). Construction and textural modification of soft surimi gel from spanish mackerel (*Scomberomorus niphonius*) by polysaccharides. Food Chem..

[41] Ma Q.-Y., Xu Q.-D., Chen N., Zeng W.-C. (2024). Gel properties of *Nicandra physalodes* (Linn.) Gaertn. seeds polysaccharides with tea polyphenols and its application. Food Chem. X.

[42] Karimi I., Mohammed L.J., Amshawee A.M., Makki N.F., Nazari K., Schiöth H.B. (2025). Mannans as multifunctional biopolymers: Structure, properties, and applications in health and industry. Polymers.

[43] Duan W., Qiu H., Htwe K.K., Wang Z., Liu Y., Wei S., Xia Q., Sun Q., Han Z., Liu S. (2023). Correlation between water characteristics and gel strength in the gel formation of golden pompano surimi induced by dense phase carbon dioxide. Foods.

[44] Wijayanti I., Singh A., Benjakul S., Sookchoo P. (2021). Textural, sensory, and chemical characteristic of threadfin bream (*Nemipterus* sp.) surimi gel fortified with bio-calcium from bone of Asian sea bass (*Lates calcarifer*). Foods.

[45] Petcharat T., Benjakul S. (2017). Effect of gellan and calcium chloride on properties of surimi gel with low and high setting phenomena. RSC Adv..

[46] Chelu M., Popa M., Ozon E.A., Cusu J.P., Anastasescu M., Surdu V.A., Moreno J.C., Musuc A.M. (2023). High-content aloe vera based hydrogels: Physicochemical and pharmaceutical properties. Polymers.

[47] Wei W., Hu W., Zhang X.-Y., Zhang F.-P., Sun S.-Q., Liu Y., Xu C.-H. (2018). Analysis of protein structure changes and quality regulation of surimi during gelation based on infrared spectroscopy and microscopic imaging. Sci. Rep..

[48] Liu J.-Y., Yoshida A., Gao Y.-L., Shirota K., Shiina Y., Noguchi E., Kuwahara K., Osatomi K. (2019). Purification and characterization of a sarcoplasmic serine proteinase from threadfin bream *Nemipterus virgatus* muscle. Food Chem..

[49] Xiong Z., Wang X., Li M., Shi T., Jin W., Li J., Yuan L., Gao R. (2023). Investigation of the enhancement mechanism of ethanol addition on the gel performance of heat-induced surimi. J. Food Eng..

[50] Gautam A.R., Benjakul S., Singh A. (2024). Transglutaminase from Asian seabass liver: Purification, characterization and cross-linking activity against natural actomyosin. Discov. Food.

[51] Dada M., Popoola P. (2024). Aloe vera hydrogel for supercooling applications: A review. Discov. Mater..

[52] Lad V.N., Murthy Z.V.P. (2013). Rheology of *Aloe barbadensis* Miller: A naturally available material of high therapeutic and nutrient value for food applications. J. Food Eng..

[53] Cunniff P. (1999). Official Methods of Analysis of AOAC International: Food Composition, Additives, Natural Contaminants.

[54] Murugan G., Nilsuwan K., Prodpran T., Ponnusamy A., Rhim J.-W., Kim J.T., Benjakul S. (2024). Active fish gelatin/chitosan blend film incorporated with guava leaf powder carbon dots: Properties, release and antioxidant activity. Gels.

[55] Ainsworth E.A., Gillespie K.M. (2007). Estimation of total phenolic content and other oxidation substrates in plant tissues using Folin–Ciocalteu reagent. Nat. Protoc..

[56] Kim D.-O., Jeong S.W., Lee C.Y. (2003). Antioxidant capacity of phenolic phytochemicals from various cultivars of plums. Food Chem..

[57] Gholkar M.S., Li J.V., Daswani P.G., Tetali P., Birdi T.J. (2021). ^1^H nuclear magnetic resonance-based metabolite profiling of guava leaf extract: An attempt to develop a prototype for standardization of plant extracts. BMC Complement. Med. Ther..

[58] Murugesan S., Buamard N., Zhou A., Zhao Y., Zhang B., Benjakul S. (2025). Improvement of gelling properties of surimi from threadfin bream (*Nemipterus* spp.) using saponin. Appl. Food Res..

[59] Laemmli U.K. (1970). Cleavage of structural proteins during the assembly of the head of bacteriophage T4. Nature.

[60] Benjakul S., Saetang J., Mittal A., Seow E.K., Singh A. (2025). Incorporation of omega-3 enriched shrimp oil nanoliposomes in threadfin bream surimi gel: Gel properties, oxidative stability, and bioavailability assessed via Caco-2 cells. Food Biosci..

